# Case Report: Series report and literature review on postoperative subdural hygroma complications following biportal endoscopic spinal surgery

**DOI:** 10.3389/fsurg.2025.1685091

**Published:** 2025-10-27

**Authors:** Guangchao Bai, Guojun Wei, Yongzhi Jian, Xiaowei Jing, Qingfeng Hu

**Affiliations:** Department of Orthopaedics, The Fourth Affiliated Hospital of School of Medicine, and International School of Medicine, International Institutes of Medicine, Zhejiang University, Yiwu, China

**Keywords:** biportal endoscopic spinal surgery, subdural hygroma, dural tear, intraspinal pressure, ischemia-reperfusion injury

## Abstract

**Background:**

Biportal Endoscopic Spinal Surgery (BESS) is a minimally invasive technique that has gained popularity for performing spinal procedures, including discectomy, decompression, and interbody fusion, in the cervical, thoracic, and lumbar regions. Whilst dural tears, epidural haematoma and transient paresthesia are well-documented complications associated with BESS, cases of subdural hygroma remain exceedingly rare. The present study presents a case series of three patients who developed postoperative subdural hygroma following BESS. An accompanying literature review was conducted in order to explore potential mechanisms and management strategies.

**Case presentation:**

The initial case pertained to a 35-year-old male patient who underwent a cervical discectomy and foraminal decompression via BESS for the treatment of cervical spondylosis. Subsequently, the patient developed a cranial subdural hygroma. The second patient, a 53-year-old male, underwent a lumbar discectomy and decompression via BESS for lumbar disc herniation with concomitant spinal stenosis. Postoperative imaging revealed extensive lumbar subdural hygroma. The third case report concerns a 74-year-old male patient who underwent BESS decompression surgery for lumbar spinal stenosis. Postoperatively, the patient developed a subdural hygroma in the lower lumbar region.

**Conclusion:**

The development of subdural hygroma following BESS may be attributed to a combination of factors, including occult dural tears, abrupt alterations in intraspinal pressure, and ischemia-reperfusion injury. In view of the potential clinical implications of this rare complication, greater awareness and monitoring is warranted in the postoperative management of patients undergoing BESS.

## 1 Introduction

Spinal subdural hygroma (SSH) is an uncommon postoperative complication, first described by Schiller et al. in 1948 in the context of hematological surgery (1). It is characterized by the abnormal accumulation of cerebrospinal fluid (CSF) within the subdural space, distinct from subdural hematoma, and may present with clinical features ranging from incidental imaging findings to acute neurological deterioration. With the widespread use of magnetic resonance imaging (MRI), recognition of SSH has increased. Typical MRI findings include crescent- or spindle-shaped fluid collections with signal intensity consistent with CSF, sometimes producing characteristic appearances such as the “flying bat” sign, which help distinguish SSH from hematoma, abscess, or lipoma (2, 3). Clinically, patients may present with postoperative back pain, radiculopathy, or even cauda equina syndrome, underscoring the need for early recognition and management (2).

Although SSH has been reported following procedures such as laminectomy, spinal fusion, and Chiari decompression, its overall incidence remains exceedingly low. The pathogenesis is not fully understood, but most reports suggest an association with intraoperative dural or arachnoid tears, allowing CSF to enter the subdural space through a ball-valve mechanism (2, 3). Interestingly, SSH has also been documented in the absence of overt dural injury, indicating that additional mechanisms may contribute to its formation.

Biportal endoscopic spinal surgery (BESS) has gained popularity as a minimally invasive alternative for cervical, thoracic, and lumbar decompression procedures. While BESS offers advantages such as improved visualization and reduced tissue trauma, the continuous irrigation required during the procedure may create unique risks for CSF-related complications (4). To date, postoperative SSH following BESS has been rarely documented. We hypothesize that factors such as occult dural tears, sustained irrigation pressure, abrupt changes in intraspinal pressure, and ischemia–reperfusion injury may contribute to the development of SSH in this surgical context.

Here, we present a case series of three patients who developed postoperative SSH following BESS, accompanied by a review of the relevant literature. Our aim is to highlight the potential mechanisms, imaging features, and management strategies of this rare complication, and to provide new insights for spinal surgeons.

## 2 Case presentation

### 2.1 Case 1

**Figure 1:**
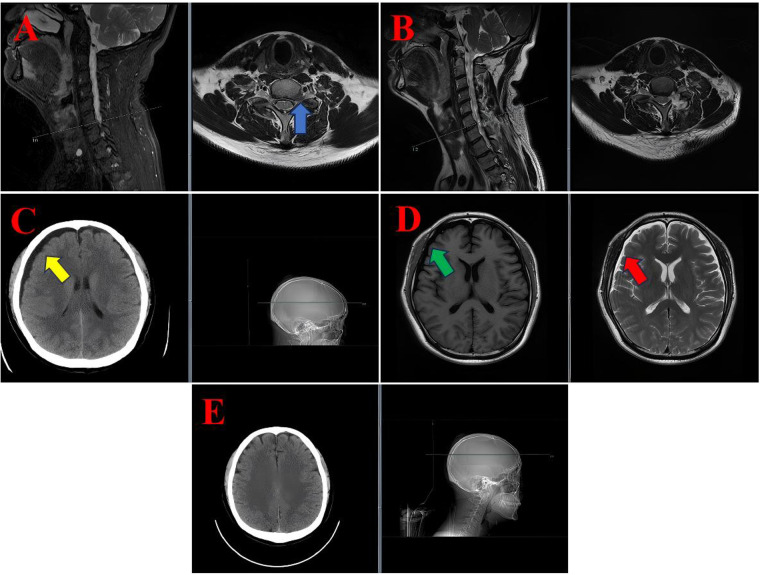
MRI and CT images of the diagnostic and postoperative course in case 1. **(A)** Preoperative cervical spine sagittal and axial T2-weighted MRI showing a left-sided disc protrusion at the C6/7 level (blue arrow). **(B)** Postoperative sagittal and axial T2-weighted MRI demonstrated no significant abnormalities at the surgical site. **(C)** Axial cranial CT scan showing a subdural hygroma (yellow arrow). **(D)** Axial cranial MRI illustrating the subdural hygroma, appearing hypointense on T1-weighted imaging (green arrow) and hyperintense on T2-weighted imaging (red arrow). **(E)** A follow-up axial cranial CT scan on postoperative day 19 demonstrated complete resolution of the subdural hygroma.

A 35-year-old Chinese male presented with a two-month history of neck pain and radiating pain in the left upper extremity. The patient had no previous medical history of chronic illness or surgical interventions. Cervical spine MRI revealed a left-sided disc herniation at the C6/7 level, accompanied by concomitant neural foraminal stenosis (see Figure 1A). The symptomatic segment was confirmed through a diagnostic left C7 nerve root block.

The patient underwent posterior cervical foraminotomy and discectomy at the C6/7 level using the biportal endoscopic spinal surgery (BESS) technique under general anesthesia. Postoperatively, a drainage tube was left in place for 48 h. The drainage volume was 78 ml during the first 24 h and 88 ml during the second 24 h, with the fluid being light red in color.

Following the removal of the drain, the patient exhibited symptoms of positional headache and dizziness. Cervical magnetic resonance imaging (MRI) revealed no abnormalities at the surgical site (see Figure 1B). However, cranial computed tomography (CT) and MRI revealed a subdural hygroma with extensive fluid accumulation (see Figures 1C,D). A neurological examination was conducted, which revealed no significant alterations in the patient's neurological status. Specifically, no focal deficits or indications of elevated intracranial pressure were observed.

A conservative management plan was initiated, encompassing the administration of intravenous mannitol (125 ml every 12 h) to reduce intracranial pressure, intravenous ceftriaxone (2 g daily) as a prophylactic measure, and oral betahistine mesylate (6 mg three times daily) to alleviate symptoms of dizziness. The patient's symptoms exhibited a progressive improvement, with near-complete resolution of headache and dizziness by postoperative day 12. A repeat cranial CT scan on day 19 confirmed complete resolution of the subdural hygroma (see Figure 1E). The patient was subsequently discharged in a stable condition.

### 2.2 Case 2

**Figure 2:**
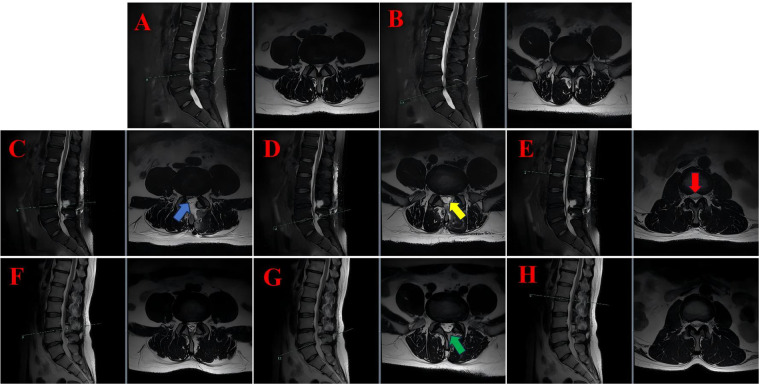
The lumbar MRI findings of case 2 illustrate the progression and resolution of postoperative subdural hygroma. **(A)** Preoperative sagittal and axial T2-weighted MRI at the L3/4 level showing disc protrusion and spinal canal stenosis. **(B)** Preoperative sagittal and axial T2-weighted MRI at the L4/5 level revealed similar findings. **(C)** On the second postoperative day, sagittal and axial T2-weighted MRI scans at the L3/4 level were performed, which revealed subdural fluid accumulation (blue arrows). **(D)** On the second postoperative day, sagittal and axial T2-weighted MRI at the L4/5 level revealed the presence of subdural fluid collection (yellow arrows). **(E)** On the second postoperative day, sagittal and axial T2-weighted magnetic resonance imaging (MRI) at the L1/2 level was conducted, which revealed superior extension of the hygroma with cauda equina compression (red arrows). **(F)** Following a period of six months, a further MRI scan was conducted, this time in the sagittal and axial planes. The scan was conducted at the L3/4 level, and revealed that the hygroma had resolved. **(G)** A six-month follow-up sagittal and axial T2-weighted MRI at the L4/5 level was conducted, confirming complete resolution of the hygroma without residual compression (green arrows). **(H)** Following a six-month period of observation, a series of MRI scans were conducted at the L1/2 level using a T2-weighted technique. These scans revealed a return to normal levels of cerebrospinal fluid distribution.

A 53-year-old Chinese male presented with a three-year history of left lower limb pain and numbness. The patient's medical history revealed a past medical history of partial thyroidectomy and gout, for which he was receiving a daily dose of 20 mg of febuxostat. Lumbar spine MRI demonstrated disc protrusion at the L3/4 and L4/5 levels with associated spinal canal stenosis (see Figures 2A,B).

Posterior lumbar laminectomy and decompression at L3/4 and L4/5 were performed using the BESS technique under general anaesthesia. A closed-suction drain was placed postoperatively and was retained for a period of 48 h. This resulted in the collection of 75 ml of serosanguinous fluid in the initial 24 h period and 60 ml in the subsequent 24 h period.

Following the removal of the drain, the patient was able to resume ambulation, with complete resolution of leg pain and significant improvement in numbness. On the second postoperative day, a routine lumbar MRI scan revealed a subdural hygroma extending beyond the surgical levels, involving the L1/2 segment and resulting in compression of the cauda equina (see Figures 2C–E). The compressed nerve roots manifested a configuration that bore a striking resemblance to a “flying bat” as depicted on axial imaging.

Notwithstanding the radiographic findings, the patient exhibited no neurological impairment, characterised by intact motor strength, preserved sensation, and normal bladder and bowel function. Following the conclusion of the treatment, the patient was discharged with scheduled outpatient follow-up. At the 6-month follow-up, repeat lumbar MRI demonstrated complete resolution of the subdural hygroma, normalization of the spinal canal dimensions, and no evidence of cauda equina compression (see Figures 2F–H). The recommendation was made for continued surveillance to be implemented.

### 2.3 Case 3

A 74-year-old male of Chinese origin presented with a two-month history of bilateral lower limb pain and intermittent claudication. The subject's medical history included hypertension, hyperlipidaemia, and coronary artery disease. The patient was prescribed amLodipine (5 mg daily) for the management of hypertension, atorvastatin (20 mg) for the regulation of cholesterol, and aspirin (100 mg) for the treatment of platelet aggregation.

**Figure 3:**
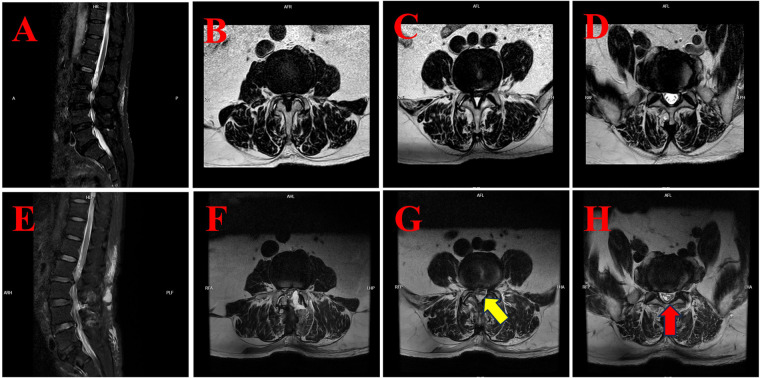
The MRI findings of case 3 illustrate the preoperative condition and postoperative development of subdural hygroma. **(A)** Preoperative sagittal T2-weighted fat-suppressed (T2WI-FS) MRI of the lumbar spine. **(B)** Preoperative axial T2-weighted MRI at the L3/4 level showing spinal canal stenosis. **(C)** Preoperative axial T2-weighted MRI at the L4/5 level showing spinal canal stenosis. **(D)** Preoperative axial T2-weighted MRI at the L5/S1 level revealed no significant abnormalities. **(E)** Postoperative sagittal T2WI-FS MRI demonstrating subdural fluid accumulation in the lower lumbar region. **(F)** Postoperative axial T2-weighted MRI at the L3/4 level with evidence of subdural fluid. **(G)** Postoperative axial T2-weighted MRI at the L4/5 level showing subdural hygroma compressing the cauda equina (yellow arrow). **(H)** Postoperative axial T2-weighted MRI at the L5/S1 level demonstrates significant subdural fluid accumulation with a compressed nerve root configuration that resembles an inverted Mercedes-Benz logo (red arrow).

Preoperative lumbar spine MRI revealed spinal canal stenosis at the L3/4 and L4/5 levels (see Figures 3B,C). Posterior decompression was performed at both levels using the BESS technique under general anaesthesia.

Postoperatively, the patient reported notable relief of pain in the left lower limb. However, he subsequently developed increased discomfort in the right buttock and posterior thigh, which was more severe than before the operation. A neurological examination was conducted, which revealed no significant changes in lower limb strength. Furthermore, perineal sensation remained unaltered. The intensity of the pain was exacerbated by movement, with the pain abating in the supine position.

Subsequent lumbar MRI revealed the presence of a subdural hygroma in the lower lumbar region, extending beyond the surgical levels to the L5/S1 segment (see Figures 3E–G). The cauda equina appeared compressed, with nerve roots at L4/5 presenting a linear configuration (yellow arrow, Figure 3G) and at L5/S1 forming a configuration resembling an inverted Mercedes-Benz logo (red arrow, Figure 3H).

The patient was treated conservatively with intravenous mannitol (125 ml every 12 h) to reduce spinal oedema and intramuscular mecobalamin (0.5 mg daily) for neurotrophic support. There was a gradual improvement in the patient's symptoms, and he was discharged with instructions for ongoing outpatient follow-up. Follow-up MRI was not performed due to patient compliance issues and significant clinical improvement; thus, clinical follow-up was used to monitor recovery.

## 3 Discussion

### 3.1 Advancements and complications of BESS technology

Biportal endoscopic spinal surgery (BESS) has attracted considerable attention in the domain of minimally invasive spine surgery over the past two decades. The BESS technique was originally developed in Argentina and first documented by De Antoni in 1996 for translaminar dural decompression using an arthroscopic system. Subsequent refinement and widespread promotion in South Korea by Jinhwa Eum led to its global adoption and rapid development (5).

The hallmark of BESS is its dual-portal approach, which offers superior visualisation and greater instrument manoeuvrability. BESS is now a standard component of surgical procedures for treating lumbar disc herniation, lumbar spinal stenosis and pathologies of the cervical and thoracic spine (6). Owing to ongoing technological innovation, the indications for its use continue to expand, encompassing intradural cysts, hematomas, abscesses, and even spinal tuberculosis. Moreover, BESS can be integrated with adjunct technologies, such as robotic navigation and 3D printing, to further enhance surgical precision and outcomes (7).

Despite its minimally invasive nature and favourable recovery profile, BESS is associated with a spectrum of postoperative complications. These include epidural hematoma, dural tears, retroperitoneal fluid collection, incomplete decompression, recurrent disc herniation, iatrogenic spinal instability, postoperative headache, anaemia, and infection (8).

The incidence of epidural hematoma has been documented as high as 24.7%, although it is noteworthy that only 1.2% of cases necessitate surgical intervention for evacuation. Risk factors have been identified, including elevated irrigation pressure and insufficient intraoperative hemostasis. Dural tears occur in 0.9%–13.2% of cases, and are often attributed to limited anatomical familiarity or poor endoscopic visualization. Postoperative anaemia, with an incidence of up to 38.9%, is generally linked to prolonged operative time and blood loss. It is well established that other complications, such as incomplete decompression, recurrent herniation, and spinal instability, are often associated with technical inexperience or inadequate preoperative planning. Postoperative back pain may be attributable to multifidus muscle injury, and headache is conceivably associated with intracranial pressure fluctuations resulting from excessive irrigation. Retroperitoneal fluid accumulation and infection are rare complications, and their incidence may be mitigated by limiting operative time and maintaining optimal irrigation parameters (9, 10).

### 3.2 Mechanisms of spinal subdural hygroma formation

The pathophysiology of spinal subdural hygroma (SSH) remains incompletely understood, but several plausible mechanisms have been proposed based on clinical and experimental observations.

#### 3.2.1 Cerebrospinal fluid leakage

The most widely accepted theory attributes SSH to cerebrospinal fluid (CSF) leakage following a breach in the arachnoid membrane. Such tears can be categorised as iatrogenic, traumatic, or spontaneous in nature, and they frequently occur in proximity to the spinal nerve root sleeves, where the dura mater is anatomically thinner and more vulnerable to disruption (11).

In addition to mechanical defects, alternative pathways for CSF translocation have been suggested. It is postulated that the cerebrospinal fluid (CSF) may be observed to migrate from the subarachnoid space into the subdural compartment. This migration may occur via perivascular channels or disrupted arachnoid layers. Thereafter, the CSF may enter the dural venous plexus and dural sinuses (12). These findings suggest that SSH may occur even in the absence of a visible arachnoid tear, particularly when normal CSF absorption or circulation pathways are impaired.

#### 3.2.2 Inflammatory response

Inflammatory processes have also been posited as a potential contributor to SSH formation. In the context of purulent meningitis complicated by subdural hygroma, histopathological studies have demonstrated the presence of inflammatory thrombi in meningeal vessels. These thrombi increase vascular permeability and promote exudate accumulation within the subdural space (13). Although postoperative inflammation is rare in degenerative spinal surgery, it has the potential to exacerbate fluid imbalance in susceptible patients.

#### 3.2.3 Pressure gradient shifts

It is hypothesised that alterations in intracranial and intraspinal pressure dynamics may exert a substantial influence on the development of SSH. The Monro-Kellie doctrine stipulates that the volume of intracranial contents-comprising brain tissue, blood, and cerebrospinal fluid-remains constant within the fixed space of the cranial cavity. A reduction in one component is known to prompt a compensatory increase in the others (14). A similar compensatory mechanism is hypothesised to occur within the spinal canal, which is rigid and bounded by inelastic bony and fibrous structures. It is hypothesised that decompression procedures, which remove part of these constraints, may allow for the redistribution of cerebrospinal fluid (CSF) into the expanded intradural space. This, in turn, may facilitate subdural fluid accumulation.

### 3.3 Case-based hypothesis of subdural hygroma pathogenesis following BESS

It is proposed that the formation of subdural hygroma (SDH) in patients undergoing BESS is attributable to three contributing mechanisms, as evidenced by clinical observations. These mechanisms include occult dural tears, abrupt shifts in intraspinal pressure, and inflammation-related permeability changes.

Firstly, it is crucial to acknowledge the significance of intraoperative dural tears in the development of SDH. In contrast to conventional open surgery, BESS employs continuous saline irrigation under constant hydrostatic pressure. In the event of an unnoticed dural defect, the pressurised fluid may dissect through the interface between the dura mater and the arachnoid membrane. This process establishes a potential space of lower resistance, which is subsequently filled with irrigation fluid until equilibrium is achieved. In support of this mechanism, a retrospective review of intraoperative video from Case 1 revealed a small, unrecognised dural tear that was not repaired during the procedure (Supplementary Video S1).

Secondly, the hypothesis is that rapid alterations in intraspinal pressure may play a pivotal role. In patients diagnosed with spinal stenosis, degenerative changes, including disc protrusion, ligamentum flavum hypertrophy, and facet joint osteophytes, contribute to restricted cerebrospinal fluid (CSF) dynamics and elevated local pressure (15). In traditional open decompression, piecemeal resection of the ligamentum flavum with Kerrison rongeurs permits gradual pressure normalization. However, BESS frequently utilises an *en bloc* resection technique (16), which, despite being less invasive, has the potential to result in sudden decompression. Moreover, upon cessation of pressurised irrigation at the conclusion of surgery, a secondary rapid decline in epidural pressure ensues. Such abrupt changes may permit the entry of cerebrospinal fluid (CSF) into the subdural space through microscopic arachnoid disruptions, as a compensatory volumetric shift.

Thirdly, it is hypothesised that postoperative ischemia–reperfusion injury may initiate a cascade of inflammatory responses, increasing meningeal permeability and facilitating fluid leakage. Following decompression in stenotic segments, reperfusion injury has been demonstrated to activate endothelial and inflammatory cells, triggering the release of cytokines such as TNF-α, IL-1, and IL-6 (17). These cytokines have been hypothesised to disrupt the integrity of the arachnoid barrier, thereby impeding the process of cerebrospinal fluid resorption and resulting in subdural fluid accumulation.

### 3.4 Imaging characteristics of spinal subdural hygroma

Magnetic resonance imaging (MRI) is the preferred modality for the identification and characterisation of spinal subdural hygroma (SSH), offering detailed visualisation of fluid collections, signal intensity, and neuroanatomical distortion.

#### 3.4.1 Signal characteristics on T1- and T2-weighted imaging

The MRI signal profile of SSH closely resembles that of cerebrospinal fluid (CSF), appearing hypointense on T1-weighted images (T1WI) and hyperintense on T2-weighted images (T2WI). For instance, in Case 1, the hygroma was observed to be hypointense on T1WI (green arrow, Figure 1D) and hyperintense on T2WI (red arrow, Figure 1D). Analogous hyperintensities were observed in Case 2 (see Figures 2C–E) and Case 3 (see Figures 3G,H).

The presence of these signal features is instrumental in differentiating SSH from subdural hematoma (SDH). In the acute phase (1–3 days), SDH typically manifests as hypointense on both T1WI and T2WI; in the subacute phase (4 days to 3 weeks), it becomes isointense or mildly hyperintense on T1WI and remains hyperintense on T2WI (18).

#### 3.4.2 Space-occupying effect and morphology

On MRI, SSH typically manifests as a crescentic or fusiform collection beneath the dura mater, exerting a mass effect on the spinal cord or cauda equina (19). This compression may result in displacement or deformation of neural structures.

The characteristic morphologies observed are influenced by anatomical tethers such as the dentate ligaments, which span laterally between the arachnoid and dura mater, and the arachnoid septum, located posteriorly in the spinal canal (20).

In this series, the compressed neural elements adopt distinct configurations, including:

1. a linear (“一”) shape (noted in Case 3),
2. a “flying bat” appearance (Case 2), and
3. an “inverted Mercedes-Benz logo” (Case 3),

which reflect directional compression across the spinal canal.

#### 3.4.3 Multisegmental involvement

A salient imaging feature of SSH is its propensity to extend across multiple spinal levels, a distinction that sets it apart from the majority of extradural lesions, which typically remain confined to a localized region (21).

1. In the first case, the hygroma extended cranially from the cervical surgical field into the intracranial subdural space.
2. In the second case, the condition propagated in an upward direction from the lower lumbar surgical area, ultimately involving the upper lumbar segments.
3. In the third case, the fluid collection extended caudally from the operative field to the lumbosacral junction.

The observed patterns indicate the potential for the spread of the phenomenon to be facilitated by the continuity of the subdural space and the pressure differentials along the neuraxis.

### 3.5 Treatment approaches and prognosis

The optimal management of spinal subdural hygroma (SSH) is contingent upon the severity of symptoms exhibited, the extent of fluid accumulation, and the presence or absence of neurological compromise.

In cases where patients are asymptomatic or present with only mild symptoms (e.g., headache, mild paresthesia), conservative treatment is generally recommended as the primary approach. Typically, the management of such cases involves a combination of interventions aimed at reducing intracranial and intraspinal pressure. These interventions include bed rest, adequate hydration, postural modifications, and the use of osmotic agents such as mannitol. In the present case series, two patients (Cases 1 and 3) exhibited a positive response to conservative therapy involving mannitol, steroids, antibiotics, and neurotrophic agents, with spontaneous resolution of the hygroma on follow-up imaging within days to weeks.

Surgical intervention is considered in patients with:

1. The presence of progressive neurological deficits (e.g., motor weakness, cauda equina syndrome) has been observed.
2. The presence of ongoing pain or symptoms that are unresponsive to medical treatment.
3. Furthermore, this is applicable in instances of substantial fluid accumulation, resulting in considerable compression of the spinal cord or nerve root.

The surgical options available for management of this condition may include decompressive laminectomy, durotomy with hygroma evacuation, or repair of dural tears if confirmed as the underlying cause of the CSF leakage during the surgical procedure. However, it is imperative to meticulously evaluate the risk-benefit ratio, given the potential for iatrogenic injury and postoperative adhesions.

The prognosis for SSH is generally favourable, especially when diagnosed early and managed appropriately. The majority of cases are known to resolve either spontaneously or with minimal intervention, as evidenced by the cases presented in this series. However, there have been reports of recurrent or chronic SSH, potentially due to unrecognised or untreated dural defects, or persistent alterations in CSF dynamics (22, 23).

Consequently, long-term follow-up is imperative. It is recommended that:

1. The utilisation of serial MRI scans is a method of monitoring resolution or recurrence.
2. The purpose of the neurological examination is to detect delayed deficits.
3. Furthermore, it is imperative to educate patients on the potential recurrence of symptoms.

The timely recognition of re-accumulation or new-onset symptoms is crucial for the implementation of effective interventions and the enhancement of long-term prognoses.

## 4 Conclusion

Spinal subdural hygroma (SSH) is an exceedingly rare but clinically significant complication following Biportal Endoscopic Spinal Surgery (BESS). Based on our observations and a review of the literature, its pathogenesis may involve multiple mechanisms. Occult dural tears can create potential pathways for irrigation fluid or cerebrospinal fluid (CSF) to accumulate within the subdural space. In addition, the continuous saline irrigation and en-bloc decompression commonly employed during BESS may precipitate abrupt alterations in intraspinal pressure, further facilitating CSF entry into the subdural compartment. Finally, local ischemia–reperfusion following decompression may trigger inflammatory cascades that disrupt the permeability of the dura–arachnoid interface, thereby contributing to hygroma formation.

The present case series highlights several distinctive features of SSH after BESS. First, the fluid collections often extended across multiple spinal segments, rather than remaining localized to the surgical level. Second, the imaging findings demonstrated characteristic morphologies such as the “flying bat” or “inverted Mercedes-Benz logo,” which may serve as useful diagnostic clues. Third, despite the extent of fluid accumulation, all three patients achieved favorable outcomes with conservative management, underscoring that SSH may be self-limiting in many cases if recognized early and monitored appropriately.

Taken together, these findings suggest that BESS-specific factors, such as continuous irrigation pressure and rapid decompression, may predispose to SSH, even in the absence of overt dural tears. Our cases provide new insights into the clinical spectrum, imaging characteristics, and prognosis of this rare complication. Heightened awareness, careful intraoperative inspection for dural injury, and diligent postoperative monitoring remain essential. Further multicenter studies are warranted to elucidate the precise mechanisms and to establish standardized preventive and therapeutic strategies for SSH in the era of endoscopic spine surgery.

## Data Availability

The original contributions presented in the study are included in the article/Supplementary Material, further inquiries can be directed to the corresponding author.

## Supplementary material

Supplementary Video S1 Shows the intraoperative identification of an occult dural tear in Case 1 during BESS cervical discectomy and decompression. The tear was located at the left C6/7 level and was not identified during the operation, which likely contributed to the subsequent development of a postoperative subdural hygroma.
